# Research on Integration of Emotion Analysis in English Modular Teaching Based on Natural Language Processing

**DOI:** 10.3389/fpsyg.2022.928883

**Published:** 2022-07-22

**Authors:** Fuxing Su

**Affiliations:** College of Foreign Languages, Shandong Technology and Business University, Yantai, China

**Keywords:** natural language, affective analysis, English module teaching, integrating emotional, Naive Bayes

## Abstract

Now, the application of intelligent technologies such as machine learning and deep learning in natural language processing has achieved good results. This article studies the integration of emotion analysis in English module teaching of natural language processing. Vocabulary is a very important part in English teaching. Learning vocabulary well can improve students’ reading ability. However, in the process of students’ learning, vocabulary is the most basic and difficult to learn. Poor vocabulary learning and insufficient accumulation will restrict students’ reading ability. Improving vocabulary teaching mode and learning methods can stimulate students’ interest in learning and effectively improve their reading ability. In the third part of the article, the neural network language model and statistical model are used to analyze the key technologies of natural language processing, and then the Naive Bayes algorithm and support vector machine model algorithm are used to optimize the data. Finally, two classes are selected for comparative experiment, then, by integrating emotional teaching into students’ classroom and analyzing students’ interest, the conclusion is that integrating emotional teaching in teaching can effectively improve students’ academic achievements, and at the same time, integrating emotional teaching in teaching can also stimulate students’ enthusiasm for learning English and effectively change students’ learning attitude.

## Introduction

In this article, a unified neural network architecture and learning algorithm are proposed, which is based on a large number of unlabeled training data to learn internal representation. This work is used as the basis for building a free and available labeling system with good performance and least computational requirements (Collobert, 2011). The purpose of this article is to develop a general-purpose natural language processor to identify clinical information in narrative reports and transform this information into structured representations containing clinical terms. Results: Without training for these four diseases, the recall rate and accuracy rate of the system were 70 and 87%, respectively. The training of query components improves the recall rate to 85% without changing the accuracy (Carol, 1994). The system of extracting structured information from natural language paragraphs has achieved great success in specialized fields. We modified the existing medical natural language processing system MedLEE to realize Genies, and made a preliminary evaluation study. Our results demonstrate the value of potential technologies for obtaining valuable knowledge from biological journals (Carol et al., 2001). In this article, we propose an emotion analysis method to find out the emotion related to a specific topic from the document, instead of dividing the whole document into positive and negative categories. In order to improve the accuracy of emotion analysis, it is very important to correctly identify the semantic relationship between emotion expression and theme (Nasukawa and Yi, 2003). The purpose of this study is to develop a method based on natural language processing. The advantage of this method is to map text to code and other relevant information, so that the coded output is suitable for effective retrieval (Carol et al., 2004). Emotion is a related problem that affects students’ learning progress and learning effect. In the past decades, people have been pursuing effective teaching methods, from grammar-translation method to communicative language teaching. Now, people begin to pay attention to emotional teaching and have achieved some good results (Zhang, 2009). The invention discloses a systematic English teaching method and a computer-aided recording medium thereof. The user evaluates by executing the test questions provided by the learning module, organizes the learning data in the learning module according to the level classification index, and the user gradually learns English using the learning data (Lin and Jiang, 2003). Through investigation, this article finds out the advantages and disadvantages of English teaching in college classroom, and analyzes the reasons for the low efficiency and long-time of college English teaching. On this basis, this article puts forward five countermeasures to change the teaching ideas, contents, methods, means and horizons, so as to change the unfavorable situation of college English teaching (Wang, 2002). Language in real communication contains many lexical chunks, and lexical chunk method conforms to the characteristics of oral discourse, which is helpful for college students to understand discourse structure and speech rules, and improve the fluency and accuracy of oral discourse (Liu, 2006). Proficiency is a goal of college English teaching. College English education should not only be the teaching of language skills, but should be the goal pursued by college English teachers. Cognition, cultural ability, subjective consciousness and emotion are all important educational factors that college English teachers need to consider (Dai, 2001). Existing research on affective computing mainly focuses on a single media, whether it is text subtitles or visual content. In this article, we discuss the learning of highly non-linear relationships between low-level features under different modes of emotion prediction (Pang et al., 2015). At present, MTV has become widely loved by people. Emotion analysis can extract emotional states included in MTV, and provides a potential and promising solution for efficient and intelligent access to MTV (Zhang et al., 2008). In this article, information features are extracted from heterogeneous inputs to express human emotions, and a hierarchical multi-modal structure based on attention is proposed, which proves the visualization of this model and explains the attention to patterns (Gu et al., 2018). Patients’ emotional/emotional state is closely related to the rehabilitation process and their health. This article presents the design and implementation of an emotion analysis module integrated in the existing telemedicine platform. The technical details of the implementation of the scheme are discussed, and the preliminary results of the accuracy and error of the scheme in actual operation are given (Kallipolitis et al., 2020). This study explores the problem of emotion measurement in the field of emotion computing, and explains the significance of discrete emotion category method and dimension method, and further puts forward dimension method based on quantitative analysis, which is the main way to solve emotion computability. Pad dimension model is of great significance to promote the research of affective computing (Ye and Mi, 2009).

## The Content and Development of English Module Teaching

### Overview of Modular Teaching Theory

Modular teaching is a new type of teaching that rationally uses modular thinking in industrialized technology. Specifically, it divides subject knowledge into individual knowledge points, and sort out the internal logic of knowledge points, recombine them into relatively independent units, integrate relevant units according to different abilities required by professional posts or technical fields, and form teaching modules, and carry out teaching by increasing or decreasing units or readjusting the mode of combination. This modular thinking is based on flexible combination, which fully realizes the integration of various post skills in the post group, which is called modular teaching.

### Module Theory at Home and Abroad

The research on module teaching in foreign countries started from the research on teaching mode, and it has been more than 30 years since then. Some colleges and universities in developed countries have formed relatively perfect teaching mode and have their own characteristics. The well-known modes are CEB teaching mode, modular teaching mode and dual system teaching mode. These three models have five common characteristics: First, these three models all adopt non-disciplinary teaching mode, but are based on ability. Secondly, the teaching process of the three modes is a complete behavior mode. Through the acquisition of information, we can work out the teaching work plan, make corresponding countermeasures, implement the teaching work plan, adjust the work quality, and finally evaluate it. Thirdly, the three modes of teaching are student-centered, in line with the principle of student-oriented. In addition, the role of teachers in the three models has changed from the traditional teaching leader to the instructor and supervisor in teaching activities. Finally, the three models attach great importance to the evaluation and control of the quality in the whole teaching process, and adopt the goal teaching method.

### Research on the Present Situation of English Teaching

In China, due to the promotion of curriculum reform, the concept of module has been widely used in English reading teaching at universities, vocational colleges, primary and secondary schools, such as English module teaching and English module teaching materials. In English teaching, modular teaching improves the teaching efficiency of English teaching. At the same time, these modules have been practiced and studied in the large-scale English teaching system. The basic method is to decompose the training of grammar, vocabulary and reading and writing ability in traditional reading teaching into teaching modules. Under this teaching mode, English knowledge learning and language application ability training are completed systematically and in modules. It not only improves the teaching effect, but also saves teaching time.

### Modular Teaching at Home and Abroad

Nowadays, English is very important for senior high school students, and there are clear regulations that students need to master vocabulary. In recent years, the requirements for vocabulary are getting higher and higher, and students memorize words by rote, but the effect is not ideal. Under the influence of proactive memory and postactive memory, especially for secondary vocational school students, vocabulary memory has become a “big problem” in the process of English learning, which not only takes a long time to remember, but also has poor effect. The role and mechanism of module teaching in vocabulary teaching of English reading has been widely studied by scholars at home and abroad. In English learning, the amount of vocabulary directly affects the mastery and application of English learners’ basic listening, speaking, reading and writing abilities. Vocabulary teaching is the key and difficult point of English teaching in secondary vocational schools.

### Modular Vocabulary Teaching Affects English Reading Ability

Scholars and teachers have studied the influence of vocabulary module teaching on reading ability from different angles, some from a specific module, and some from a universal module teaching method. When discussing the improvement strategies of English reading teaching, scholars point out that the process of English reading teaching is the process of readers’ language communication and thinking cognition, and students’ non-linguistic knowledge and linguistic knowledge play an important role in the application of interactive reading teaching mode. This teaching mode can guide senior high school students to strengthen their understanding of the text from the whole to the details, and make reading conform to the application of language.

## Key Technologies of Natural Language Processing

### Overview of the Language Model

Natural language processing is a sub-problem in the field of artificial intelligence. Its purpose is to make computers understand human language efficiently and complete specific tasks instead of human beings. Nowadays, there are two popular processing methods: rule-based method and statistics-based method. The former is based on the rules expressed in language use, while the latter is based on the statistical analysis of large-scale data to find potential rules to achieve data processing. Therefore, having large-scale and high-quality data is particularly important for natural language processing.

The task of language model is to assign a probability value to sentences. Language model plays an important role in natural language processing. For example, machine translation system can score its translated sentences through language model. With the chain rule, we write the probability of a given word sequence *w*_*1:n*_ as formula (1):


(1) $$P\,\left(w_{1:n}\right)=P\,\left(w_{1}\right)\,P\,\left(w_{2}|w_{1}\right)\,P\,\left(w_{3}|w_{1:2}\right)\,\cdots \,\cdots \,P\,\left(w_{n}|w_{1:n-1}\right)$$

The key to determining the predicted words in the sequence lies in all the previous words. Then the last word in the sentence depends on the first 1 words, which makes the model parameters very large and makes modeling difficult. Formula (2) assumes that the words appearing at time T + 1 are only related to the previous K words:


(2) $$P\,\left(w_{t+1}|w_{1:t}\right)\approx P\,\left(w_{t+1}|w_{t-k+1:t}\right)$$

Then the sentence probability estimation formula is formula (3):


(3) $$P\,\left(w_{1:n}\right)=\Pi_{i=1}^{n}\,P\,\left(w_{i}|w_{i-k:i-1}\right)$$

When the current word is only related to the previous word, we call the model a binary language model. Although Markov’s hypothesis has obvious errors, it greatly reduces the complexity of the model. Training language model is a statistical model of corpus, and then estimates formula (4) *P*(*w*_*i*_|*w*_*i*−*k*:*i*−1_). Assuming that,


(4) $$c\,\left(w_{i-k:i-1}\right)$$

Represents a count of the number of occurrences of *w*_*i–k:i–1*_ in the corpus, formula (5) is obtained from the maximum likelihood estimation:


(5) $$P_{M\,L\,E}\,\left(w_{i}|w_{i-k:i-1}\right)=\frac{c\,\left(w_{i-k:i}\right)}{c\,\left(w_{i-k:i-1}\right)}$$

The probability of testing data is the most commonly used method to evaluate models. Given the test data set and the language model LM that assigns probability to sentences, the confusion degree is calculated as formula (6):


(6) $$P\,e\,r\,p\,l\,e\,x\,i\,t\,y=2^{-\frac{1}{n}\,\sum \begin{array}{c} n\\ i=1 \end{array}\,\log_{2}L\,M\,\left(w_{i}\,w_{1:i-1}\right)}$$

### Neural Network Language Model

The input of the neural network is a context word *w*_*1:k*_, and the output is the probability distribution of the k + 1th word *w*_*k+1*_. According to matrix multiplication, each word embedding w and the word vector *v*(*w*) one to one, and then the concatenated word vectors as input to the neural network as Eq. (7):


(7) $$x=\left[v\,\left(w_{1}\right);v\,\left(w_{2}\right);v\,\left(w_{3}\right);\cdots ;v\,\left(w_{k}\right)\right]$$

Then input to the multilayer perceptron with one or more hidden layers to get formula (8), formula (9):


(8) $$h=g\,\left(W_{1}\,x+b_{1}\right)$$

(9) $$y_{h\,a\,t}=s\,o\,f\,t\,\max\left(W_{2}\,x+b_{2}\right)$$

*y*_*hat*_ for the |*V*| dimensional vector represents the probability distribution of the predicted words. Non-linear neural network language model solves some problems in traditional language models. Neural network language model can obtain a longer context information by only adding a small number of parameters, without manual design of retreat rules, and has stronger generalization ability.

Assuming that the matrix *A*_*nm*_ is a word-document matrix, the matrix element *a*_*wd*_ represents the statistical value of the word w in the document d, the matrix *A*_*nm*_ is decomposed into three matrices multiplied according to the SVD technology, such as formula (10):


(10) $$A_{n\,m}=U_{n\times p}\,\sum_{p\times p}V_{p\times m}^{T}$$

The matrix U is known as a left-singular matrix. Based on the co-occurrence relationship of words in the same document, the matrix gives the relationship between words and subjects, which is an important matrix related to semantic analysis.

Neural probabilistic language models are proposed after the affine layer and around the layer. And replace the maximum probability with 1 and the other with 0. The continuous word bag method uses surrounding words to predict central words, and the input of the model is the vector sum of the single heat vector of the surrounding words, such as Eq. (11):


$$A_{n\,m}=U_{n\times p}\,\sum_{p\times p}V_{p\times m}^{T}\,x$$

(11) $$=\left(v\,\left(w_{t-2}\right)+v\,\left(w_{t-1}\right)+v\,\left(w_{t+1}\right)+v\,\left(w_{t+2}\right)\right)$$

Then take the mean of the context N word vector to obtain the formula (12):


(12) $$h=\frac{1}{N}\,\left(x\,W_{\left|V\right|\times d}\right)$$

|*V*| represents the size of the vocabulary, as the dimension of the word vector. Then map it to a vector with dimension P such as the formula (13):


(13) $$y=h\,W_{d\times \left|V\right|}$$

Softmax function is a normalized function, which is mainly used in the operation of probability theory in mathematics. It can transform one dimension vector z into another vector, so that its value is in a controllable range. This function is more generalized in multi-classification problems. The probability distribution of the target word is finally obtained by the softmax function, such as the formula (14):


(14) $$y_{h\,a\,t}=\frac{1}{n}\times \left|\frac{y_{i}}{\sum_{i=1}^{n}\exp\left(y_{i}\right)}\right|$$

Eq. (15) is the mean of the input vector of the hidden layer.


(15) $$h=\frac{1}{n}\,\sum \begin{array}{c} n\\ i=1 \end{array}\,v_{i}$$

### Statistical Model

The information gain algorithm and the information gain ratio algorithm are based on the concept of entropy. Entropy is used to measure the uncertainty in random variables. The larger the value of the entropy represents the greater the uncertainty of the random variables, such as formula (16):


(16) $$H\,\left(X\right)=-\sum \begin{array}{c} n\\ i=1 \end{array}\,p_{x_{i}}\,\log p_{x_{i}}$$

Conditional entropy is the way to measure the uncertainty of the X. As shown in formula (17):


(17) $$H\left(X|Y\right)=-\sum \begin{array}{c} n\\ i=1 \end{array}p_{y_{i}}H\left(X|Y=y_{i}\right)$$

among, *py_i_* = *P*(*Y* = *y*_*i*_),*i* = 1,2,⋯⋯*n* information gain is defined as the difference between the entropy of a random variable X and the conditional entropy under a given random variable Y: as shown in formula (18):


(18) g⁢(D,A)=H⁢(D)-H⁢(D|A)

*g*(*D*,*A*) represents the information gain of the dataset D for a given feature A.

In order to simplify the feature selection, the information gain ratio algorithm is proposed, as shown in formula (19) and formula (20):


(19) $$g_{r}\,\left(D,A\right)=\frac{g\,\left(D,A\right)}{H_{A}\,\left(D\right)}$$

(20) $$H_{A}\,\left(D\right)=\sum \begin{array}{c} n\\ i \end{array}\,\frac{\left|D_{i}\right|}{\left|D\right|}\,\log\frac{\left|D_{i}\right|}{\left|D\right|}$$

Where *n* represents the number of feature A values. The CART algorithm uses the Gini index as a feature selection algorithm: as shown in formula (21):


(21) $$G\,i\,n\,i=\sum \begin{array}{c} k\\ i=1 \end{array}\,p_{k}\,\left(1-p_{k}\right)=1-\sum \begin{array}{c} k\\ i=1 \end{array}\,p_{k}^{2}$$

CART algorithm is a kind of implementation of decision tree, CART algorithm is a binary recursive normalization function, the current sample is divided into two sub-samples, so that each generated non-leaf node has two branches. Gini refers to the Gini coefficient, which is a proportional value.

### Naive Bayes Algorithm

Naive Bayesian classification is based on Bayes’ theorem and conditional independence assumptions. Naive Bayesian classification methods have simple principles and insensitive to missing data.

The naive Bayesian classification method estimates the probability of the text belonging to each category from the given text characteristics, and then classifies the text into the category with the highest probability. Assuming that the training set has a species type *c*_1_,*c*_2_,⋯⋯,*c*_*k*_ ∈ *C*, the probability that the feature word of the text has an *w*_1_,*w*_2_,⋯⋯,*w*_*n*_ Bayesian classifier is expressed as the formula (22):


(22) $$c_{i}=\arg\max\left(p\,\left(c_{i}|w_{1-n}\right)\right),c_{i}\in C$$

*p*(*c*_*i*_|*w*_1−*n*_) indicates the probability of belonging to a given feature word *w*_*1–n*_, and obtains the formula derived from Bayes’s theorem (23):


(23) $$p\,\left(c_{i}|w_{1-n}\right)=\frac{p\,\left(w_{1-n}|c_{i}\right)\,p\,\left(c_{i}\right)}{p\,\left(w_{1-n}\right)}$$

The naive Bayesian classifier learns the joint probability distribution of category and feature word 1 through the training set, and then finds the *c*_*i*_ with the largest posterior probability. Naive Bayes is called “naive” because the method is based on conditional independence assumptions, as shown in formula (24):


(24) $$p\,\left(w_{1-n}|c_{i}\right)=\Pi_{j=1}^{n}\,p\,\left(w_{j}|c_{i}\right)$$

The conditional independence assumption means that the characteristic words are independent of each other under a given condition. According to the experience of the real-life species, we know that the assumption is not accurate, and that there are dependencies between the words in the statements. But this assumption greatly reduces the model parameters, sacrifices accuracy but makes the model easier to train. Through emotion control and probability statistics, different emotions will cause different results. Establishing a probability model between different emotions and results can better analyze the causes of results. In this article, Naive Bayes probability and statistical model is used to analyze the relationship between the results caused by different emotions, and the re-nature of emotions in English word teaching is obtained.

### Support Vector Machine

Support vector machine is a commonly used binary classification model. In order to make the hyperplane dividing the sample more robust, it selects the best partition hyperplane on the sample feature space. For the linear separable dataset, the hard interval.

Suppose that $\left\{{\left(x_{i},y_{i}\right)}_{i=1}^{N},x_{i}\in R^{n},y_{i}\in \left\{-1,+1\right\}\right\}$ is linear and separable for a given dataset, and that there is a hyperplane to correctly divide the dataset, as shown in formula (25):


(25) $$y_{i}\,\left(w^{T}\,x_{i}+b\right)>0$$

When *w^T^x*_*i*_ + *b* > 0, will *y*_*i*_ = + 1; when *w^T^x*_*i*_ + *b* < 0, will *y*_*i*_ = −1. We define the support vector as the shortest point of distance from the hyperplane and the distance from the support vector to the hyperplane, as shown in formula (26):


(26) $$m\,\arg i\,n=\min_{x_{i}}\left(\frac{1}{||w||}\,\left|w^{T}\,x_{i}+b\right|\right)$$

The SVM maximizes the interval to find the optimal partition hyperplane, as shown in formula (27):


(27) $$\max_{w,b}\min_{x_{i}}\left(\frac{1}{||w||}\,\left|w^{T}\,x_{i}+b\right|\right)$$

Thus we have translated the classification problem into optimization problems such as Eq. (28):


(28) $$\left\{ \begin{array}{c} \max_{w,b}\frac{1}{||w||}\,\min_{x_{i}}\left(y_{i}\,\left(w^{T}\,x_{i}+b\right)\right)\\ s.t.y_{i}\,\left(w^{T}\,x_{i}+b\right)>0 \end{array}\right.$$

Let $\min_{x_{i}}\left(y_{i}\,\left(w^{T}\,x_{i}+b\right)\right)=1$ exist for a given hyperplane parameter w, b then the optimization problem is transformed into formula (29):


(29) $$\left\{ \begin{array}{c} \min\frac{1}{2}\,w^{T}\,w\\ s.t.y_{i}\,\left(w^{T}\,x_{i}+b\right)\geq 1 \end{array}\right.$$

By solving this convex quadratic optimization problem, the optimal partition hyperplane is obtained.

## Experiments and Experimental Results

### Research Methods

First, questionnaires were distributed to six science classes in a middle school, and then the questionnaires were collected for data analysis. Finally, two classes with similar conditions were selected through the significant difference test. When the two classes are selected, class is selected as the control class and class is the experimental class. On the basis of learning reference and related literature, combined with teaching practice, interview, test, questionnaire and controlled experimental method are used to carry out English emotion teaching research in high school. First, the pre-experimental test was conducted, and the questionnaire and interview method were selected for students to analyze the current situation of students’ interest and status in English learning. Then, one of them is randomly selected as the experimental class, in the teaching process, more emotional motivation, music rendering, picture display, performance experience, classroom teaching language; in the other class meeting, the traditional teaching mode is adopted.

Through the experiment, the role of emotion teaching is verified by post testing the students of two classes. In the specific experiment process, the pre-test and post-test of the English survey of high school students are mainly set. The main purpose of the pretest is to verify whether there is a large difference in the questionnaire of the two selected classes, so as to ensure that the two classes can investigate under the same premise and ensure the comparability of the subsequent data. The post-experimental test is mainly to analyze the research content through the data difference between the experimental class and the control class. To ensure the validity and reliability of the before and after tests, the same test time and the same scoring criteria were used.

### Implementation of Control Class and Experimental Class

The teaching mode of the control class is very simple, the students preview the teacher, and finally assign the homework. In classroom teaching, the traditional way is still “indoctrination”

teaching, mainly taught by the teacher, and reciting the key contents and chapters.

In the experimental class, teachers should change the traditional teaching methods. Use of new electronic engineering and information technology more. Create situations to provide students with a broader information platform and knowledge. In the teaching process, teachers should use the emotional incentive method. Through the creation of situational English language application ability to cultivate students’ English language application ability. Therefore, there will be certain differences between classroom discipline and other classes. For example, in the process of asking students to “listen,” In the classroom, to avoid the sound affecting the students’ learning, keep quiet; to ask the students to “say,” it can be an individual communication, it can also be a group communication, the students should be encouraged to communicate fully, encourage students to express their opinions bravely; ask students to “read,” let students deepen their understanding of the learning content by reading or reading, give students some space; in the requirement to “write,” teachers should supervise the independent completion of the students, conduct mutual evaluation or encourage students to show their results to other students, take questions from other students.

### Analysis of the Pre-experimental Test Data

To compare whether the initial test scores of the experimental class and the control class have changed greatly, a single sample *t*-value test is carried out here, and the data are shown in Tables 1, 2.

**TABLE 1:** Group statistics (previous test).

Group	*N*	Mean	St. error	St. dev. mean
Exp-class	37	87.62	8.09	1.34
Con-class	37	88.7	8.12	1.33

**TABLE 2:** Independent sample testing (previous test).

Hypoth.	*F*	Sig.	*t*	df	Sig. bilateral	Mean	Error
Equal.var	0.17	0.89	0.57	72	0.56	1.08	1.88
Var.var	0.23	0.53	0.57	71.99	0.57	1.09	1.87

As can be seen from this table, the average experimental class score was 87.62, compared with 88.70 in the control class. Whether this value reaches a statistical difference is analyzed in the table below.

This analysis showed that at the significance level of 0.05, the *P*-test for independent sample T score was 0.56, greater than 0.05. Therefore, there is no significant difference between the pretest scores of the experimental class and the control class. It shows that the two classes had the same learning level before the experiment. A total of 74 questionnaires were distributed to two classes, with 37 students in each class. The valid volume is 74, the efficiency is 100%, and the ratio of men and women in the two classes is similar. In the interview, 20 copies were distributed to 10 teachers and 10 students, and 20 copies were recovered, with an efficiency of 100%. Among them, 10 teachers were of different ages, professional titles and positions, and the male to female ratio of 10 students was 1:1. DF is the abbreviation of degree of freedom, which is the value that can be randomly changed when calculating data. The number of other independent quantities used to calculate a certain amount of data is often used in sampling distribution.

**FIGURE 1:**
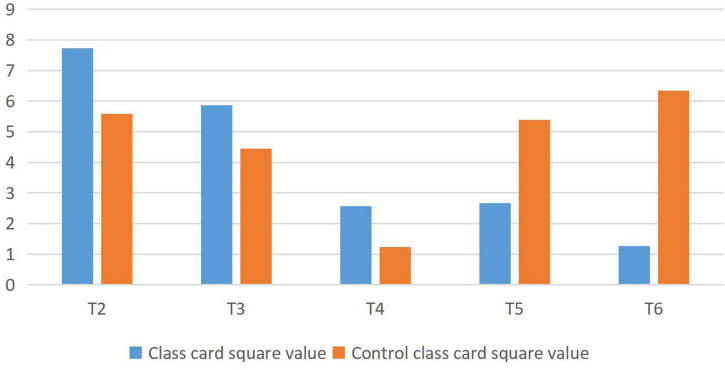
Analysis of students’ learning interest (previous test).

The analysis of students’ interest in learning is shown in Figure 1. T2 means: I like English class; T3 means: I like the teacher’s teaching method; T4 says: my teacher will communicate with students in the process of teaching; T5 means: I know what emotional teaching is; T6 means: I think teachers will increase my interest when communicating with my feelings. Chi-square values of <χ^2^ < P0.01 at P0.05 indicate significant differences, and extremely significant differences when χ^2^ > P0.01.

From the above data analysis, it can be seen that the students in the experimental class and the control class are not active in English learning before affective analysis.

**FIGURE 2:**
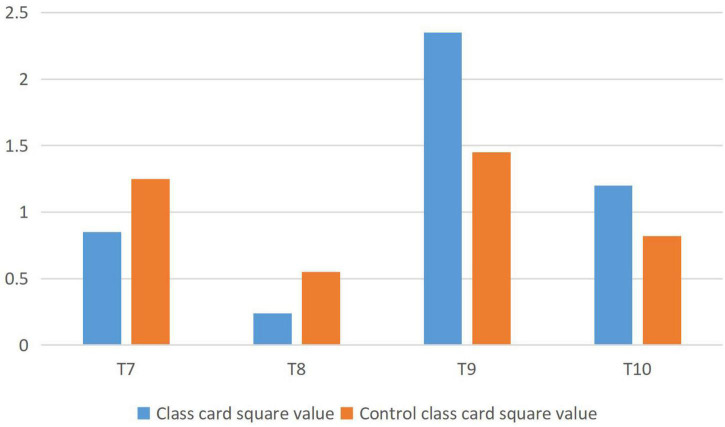
Analysis of students’ learning attitude (pretest).

The analysis of students’ learning attitude is shown in Figure 2. T7 represents: I think teachers will pay attention to students’ feelings during teaching; T8 represents my attitude toward emotional teaching in teaching; T9 represents: I think teachers’ pay attention to the application of emotional teaching; T10 means I am willing to consume more emotional teaching; when P0.05 < χ^2^ < P0.01, when χ^2^ > P0.01.

From the above data analysis, it can be seen that there is little difference between the students in the experimental class and the control class toward English learning before the experiment.

### Analysis of Post-test Data

After a semester of emotion teaching, a second data collection was conducted for the previous students. To compare whether there were significant differences between the experimental class and the control version, independent samples were also tested, as shown in Tables 3, 4.

**TABLE 3:** Group statistics (post-test).

Group	*N*	Mean	St. error	St. dev. mean
Exp-class	37	92.38	5.33	0.87
Con-class	37	89.11	7.68	1.25

**TABLE 4:** Independent sample testing (post-test).

Hypoth.	*F*	Sig.	*t*	df	Sig. bilateral	Mean	Error
Equal.var	5.65	0.21	2.14	72	0.36	3.27	1.52
Var.var	5.23	0.2	2.14	64.48	0.36	3.27	1.52

As can be seen from the table, the mean experimental class score was 92.38, compared with 89.11 in the control class. Whether this value reaches a statistical difference is analyzed in the table below.

**FIGURE 3:**
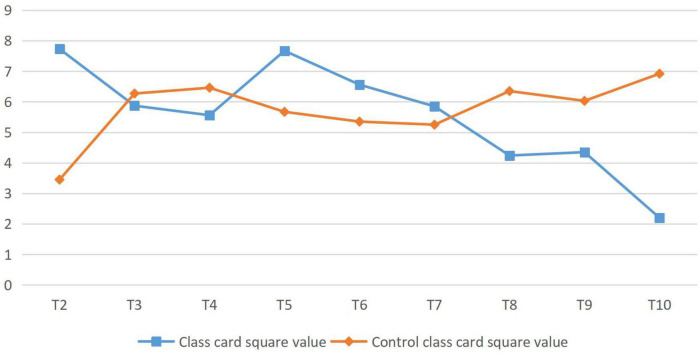
Analysis of students’ learning interest and attitude (posttest).

This analysis showed that at the significance level of 0.05, the *P*-value of the independent sample *t*-test for the pretest score was 0.036, greater than 0.05. Therefore, there were significant differences between the experimental class and the control class, and the significant difference between the experimental classes was higher than that of the control class. According to data analysis, the proportion of options in the control class was basically the same as in the previous test, while the experimental class changed significantly. Key data were specially selected for comparison, as shown in Figure 3. *T* value is the data standard of *t*-test, which is mainly used in normal distribution with small sample size and unclear population standard deviation. *T* value can compare the difference between two averages.

As can be seen from the data analysis in Figure 3, T6 showed extremely significant differences and T2 and T5.T3, and T4 did not change much. Therefore, after the experiment, the students in the experimental class and the control class’ interest in English learning changed greatly, and the students in the experimental class’ interest in English learning increased significantly. T8 and T10 showed extremely significant differences, T9 showed significant differences and little change in T7. Therefore, after the experiment, the attitude between the experimental class and the control class students toward English learning has changed greatly, and the attitude of the experimental class students toward English learning has improved significantly. The ordinate in Figure 3 represents the chi-square value of the post-test data of the analysis of students’ learning interest and attitude in the experimental class and the control class.

### Analysis of Emotional Teaching Mode

To compare whether there were significant differences in the measured scores before and after the experimental class, the paired sample *t*-test was also performed, see Tables 5, 6 below.

**TABLE 5:** Pair sample statistics (experimental class).

Group	*N*	Mean	St. error	St. dev. mean
Pre-score	37	87.62	8.09	1.34
Post-score	37	92.38	5.33	0.87

**TABLE 6:** Pair sample test (experimental class).

Group	Mean	St. error	St. dev. mean	*t*	df	Sig.
Pre-score	8.76	8.09	1.34	0.57	72	0.56
Post-score	4.75	4.41	0.72	6.57	36	0.01

It can be concluded from Table 6 that at the significance standard is 0.05, the *P*-value of the test value of the experimental class is 0, which is smaller than 0.05, so the score gap between the experimental class is large, and the post-test data is higher.

The analysis of the before and after the test scores in the control class is shown in Tables 7, 8.

**TABLE 7:** Pair sample statistics (control class).

Group	*N*	Mean	St. error	St. dev. mean
Pre-score	37	88.7	8.12	1.33
Post-score	37	89.11	7.68	1.25

**TABLE 8:** Pair sample test (control class).

Group	Mean	St. error	St. dev. mean	*t*	df	Sig.
Pre-score	0.81	7.68	1.25	2.14	72	0.36
Post-score	0.45	2.14	3.52	1.15	36	0.27

**FIGURE 4:**
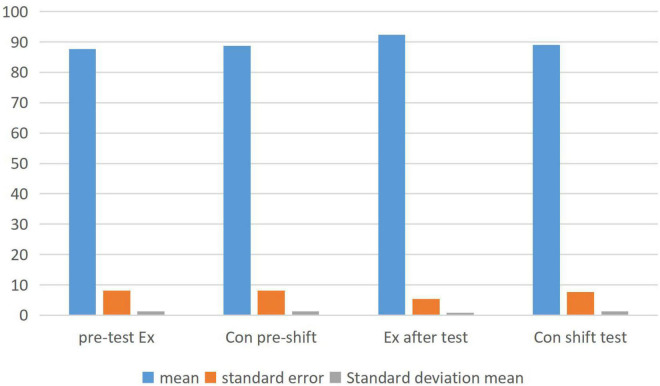
Comparison of sample statistics between experimental and control classes.

**FIGURE 5:**
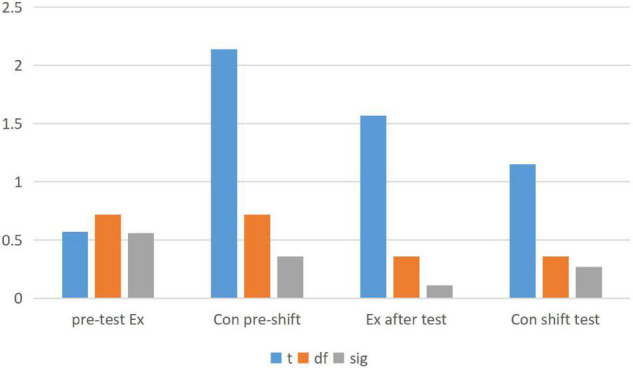
Comparison of the sample test values between the experimental classes and the control classes.

As shown from the above table. Therefore, there is not much change in the measured data scores before and after the control class. The sample statistics of the two classes are compared in Figure 4 and paired samples are compared in Figure 5.

In the analysis of Figures 4, 5, we concluded that emotion teaching can improve students’ academic performance. In the questionnaire, we also found that emotional education can stimulate students’ enthusiasm to learn English and greatly improve students’ learning attitude. However, in the process of implementing emotion teaching, we need to start from both teachers and students. From the analysis of the data of the two classes in Figure 5, it is found that the Chi-square value of the post-test of the experimental class is not so high, while the *T*-value of the pre-test of the other class is quite different. Which shows that English module teaching combined with emotion analysis is more easily accepted by students.

## Conclusion

This article combines quantitative and qualitative methods by using the model calculation method of quantitative natural language processing technology and setting up the two classes. Through the analysis of data and data, the following conclusions are obtained: First, the English module teaching integrating emotion analysis can indeed cultivate students’ interest in learning English and enhance their subjective initiative. The design before class, during and after class can help students improve the teaching quality; emotional teaching can maximize students’ confidence. This benign attribution way can help students overcome difficulties and enhance their subjective initiative; third, the emotional teaching content can enable students to better understand English regional culture, expand international vision and integrate foreign culture. Sample limitations: Samples are very important in scientific research. Whether the selected sample objects are representative and whether the sample size is large enough determines the scientific and universal research conclusions. The survey in this study is conducted in a school with limited sampling. Although it can reflect some of the current situation and problems of students’ English module learning, it cannot fully reflect the current situation of all students’ English vocabulary learning and reading ability. In the next research, we should scientifically set the scope and capacity of sample selection, break through the barrier of being limited to the teaching place, expand the research sample, and get more convincing conclusions.

## Data Availability Statement

The original contributions presented in the study are included in the article/supplementary material, further inquiries can be directed to the corresponding author/s.

## Author Contributions

The author confirms being the sole contributor of this work and has approved it for publication.

## Conflict of Interest

The author declares that the research was conducted in the absence of any commercial or financial relationships that could be construed as a potential conflict of interest.

## Publisher’s Note

All claims expressed in this article are solely those of the authors and do not necessarily represent those of their affiliated organizations, or those of the publisher, the editors and the reviewers. Any product that may be evaluated in this article, or claim that may be made by its manufacturer, is not guaranteed or endorsed by the publisher.

## References

[B1] Carol F. (1994). A general natural-language text processor for clinical radiology. *J. Am. Med. Inform. Assoc.* 2 161–174. 10.1136/jamia.1994.95236146 7719797 PMC116194

[B2] Carol F. Lyudmila S. Yves L. (2004). Automated Encoding of Clinical Documents Based on Natural Language Processing. *J. Am. Med. Inform. Assoc.* 11 392–402. 10.1197/jamia.M1552 15187068 PMC516246

[B3] Carol F. Pauline K. Yu H. (2001). GENIES: a natural-language processing system for the extraction of molecular pathways from journal articles. *Bioinformatics* 17:S74. 10.1093/bioinformatics/17.suppl_1.S74 11472995

[B4] Collobert R. (2011). Natural Language Processing (almost) from Scratch. *J. Mach. Learn. Res.* 1 2493–2537.

[B5] Dai Z. (2001). Functions of college English teaching in quality based education. *Foreign Lang. World* 12 34–67.

[B6] Gu Y. Yang K. Fu S. (2018). Multimodal Affective Analysis Using Hierarchical Attention Strategy with Word-Level Alignment. *Proc. Conf. Assoc. Comput. Linguist. Meet* 23 132–156. 10.18653/v1/P18-1207 30505068 PMC6261375

[B7] Kallipolitis A. Galliakis M. Menychtas A. (2020). Affective analysis of patients in homecare video-assisted telemedicine using computational intelligence. *Neural Comput. Appl.* 32 17125–17136. 10.1007/s00521-020-05203-z

[B8] Lin K. S. Jiang W. (2003). Step-by-step english teaching method and its computer accessible recording medium. *U.S. Pat. Appl.* 23 34–78.

[B9] Liu J. Y. (2006). Lexical Chunks and College English Teaching. *Shandong Foreign Lang. Teach. J.* 43 26–28.

[B10] Nasukawa T. Yi J. (2003). “Sentiment analysis:Capturing favorability using natural language processing. International conference on Knowledge Capture”. *Proceedings of the 2nd International Conference on Knowledge Capture (K-CAP 2003), October 23-25, 2003* (Sanibel Island, FL:DBLP) 10.1145/945645.945658

[B11] Pang L. Zhu S. Ngo C. W. (2015). Deep Multimodal Learning for Affective Analysis and Retrieval. *IEEE Trans. Multimed.* 17 2008–2020. 10.1109/TMM.2015.2482228

[B12] Wang Q. (2002). Reasons for ineffective college English teaching and relevant countermeasures. *Foreign Lang. World* 2 145–161.

[B13] Ye L. Mi T. L. (2009). Beijing. The Analysis of PAD Emotional State Model Based on Emotion Pictures. *J. Image Graph.* 3 e16–e79.

[B14] Zhang J. (2009). Analysis of affective factors and corresponding solution in English teaching. *Lib. Arts Lvrs. Educ. Teach. Ed*. 1, 4–5.

[B15] Zhang S. Huang Q. Qi T. (2008). “Personalized MTV Affective Analysis Using User Profile,” in *Proceedings of the 9th Pacific Rim Conference on Multimedia: Advances in Multimedia Information Processing*, 15. (Berlin: Springer), 331–346. 10.1007/978-3-540-89796-5_34

